# Study on Frost Resistance and Interface Bonding Performance through the Integration of Recycled Brick Powder in Ultra-High-Performance Concrete for Structural Reinforcement

**DOI:** 10.3390/ma16216999

**Published:** 2023-11-01

**Authors:** Yike Zhang, Ali Raza, Muhammad Umar, Yang Chen, Chengfang Yuan

**Affiliations:** College of Civil Engineering, Zhengzhou University, Zhengzhou 450001, China; qq1057438040@126.com (Y.Z.); m.umar@gs.zzu.edu.cn (M.U.); cy292021@163.com (Y.C.)

**Keywords:** recycled brick powder, UHPC, freeze–thaw cycle, interface bonding strength, bonding mechanism

## Abstract

This study aims to address the issues posed by frost damage to concrete structures in cold regions, focusing on reinforcement and repair methods to increase the service life of existing structures instead of costly reconstruction solutions. Due to the limitations of conventional concrete in terms of durability and strength, this research focused on ultra-high-performance concrete (UHPC) by replacing part of the cement with recycled brick powder (RBP) to strengthen ordinary C50 concrete, obtaining UHPC-NC specimens. Mechanical tests investigated the bonding performance of UHPC-NC specimens under various conditions, including interface agents, surface roughness treatments, and freeze–thaw after 0, 50, 100, and 150 cycles with a 30% replacement rate of RBP. Additionally, a multi-factor calculation formula for interface bonding strength was established according to the test data, and the bonding mechanism and model were analyzed through an SEM test. The results indicate that the interface bonding of UHPC-NC specimens decreased during salt freezing compared to hydro-freezing, causing more severe damage. However, the relative index of splitting tensile strength for cement paste specimens showed increases of 14.01% and 14.97%, respectively, compared to specimens without an interface agent. Using an interface agent improved bonding strength and cohesiveness. The UHPC-NC bonding model without an interfacial agent can be characterized using a three-zone model. After applying an interfacial agent, the model can be characterized by a three-zone, three-layer bonding model. Overall, the RBP-UHPC-reinforced C50 for damaged concrete showed excellent interfacial bonding and frost resistance performance.

## 1. Introduction

In cold regions, frost damage seriously impacts the safety and durability of concrete structures [1]. Compared with demolition and reconstruction, the scheme of identification, evaluation, repair, and reinforcement of frost-damaged concrete structures is more in line with the requirements of social development and environmental and natural resource protection than reconstruction [2,3]. Therefore, concrete repair and reinforcement are required to extend the durability of concrete [4,5,6].

However, conventional concrete still has limitations, including low durability and strength. These limitations and disadvantages restrict its use as a material for repair purposes [7,8,9]. While ultra-high-performance concrete (UHPC) has attracted increasing attention across various sectors of the construction sector in recent years, its dense microstructure significantly increases the durability and sustainability of concrete structures [10]. Moreover, UHPC has an outstandingly high compressive strength, making it highly valuable for various construction applications. The minimum compressive strength of UHPC has been reported to be between 160 and 180 MPa [11,12], rendering it highly valuable for various construction applications, particularly in maintenance and reinforcement engineering [13,14,15].

However, the high costs and environmental impacts of UHPC due to its raw materials pose challenges. Notably, the cement industry alone contributes to approximately 5–7% of global CO_2_ emissions [16,17,18]. To address this issue, researchers partially substitute OPC with alternative recycled materials [19,20,21]; this can significantly reduce the costs and carbon emission reduction challenges. Adopting cost-effective, eco-friendly alternatives with similar properties makes UHPC production more sustainable, economically feasible, and environmentally friendly [22]. Relevant research studies have demonstrated that recycled brick powder (RBP) is emerging as a promising alternative due to its high SiO_2_ and Al_2_O_3_ content, micro-aggregate properties, volcanic ash-like characteristics, alignment with auxiliary cementitious materials, and ability to be utilized as supplementary cementitious materials [23,24,25]. RBP particles are spherical in shape and size, which increases its pozzolanic activity and specific surface area. A reduction in the particle size of RBP accelerated the early hydration reaction and also decreased the setting time [26,27]. Lin [28] and Xue [29] studied the strength properties of cement-based materials using RBP with different substitution rates. Utilizing RBP as a partial substitution for cement resulted in a reduction in compressive strength. Meanwhile, the study by Gonçalves et al. [30] demonstrated that substituting 20% of cement with RBP allowed the material to fulfill strength requirements; the decrease in the proportion of macropores may be caused by the addition of RBP.

However, previous experimental studies have primarily focused on the interface bonding performance of UHPC with existing concrete in room-temperature and freeze–thaw environments [31], and reinforced specimens have shown positive bonding results in splitting, diagonal shear, three-point bending, straight shear, and oblique shear tests without interface roughness conditions [32,33,34]. Additionally, Mingzhe An et al. [24] and Liang et al. [35] investigated the damage mechanisms of UHPC when exposed to freeze–thaw cycles in a salt solution, focusing on rehydration effects. This study focuses on the challenges UHPC may face in corrosive environments and cold climates, contributing to a broader understanding of UHPC performance in challenging conditions. Further investigation is needed, especially considering the effects of interface roughness. Additionally, the impact of RBP as a partial cement substitution on bonding behavior remains an open question.

He Yan et al. [36] evaluated the mechanical characteristics of concrete binders and the effects of the interface roughness of new and existing concrete and found that an interface binder significantly improved the strength of new and existing concrete and the bonding strength. Liao Zhaoqian and Wang et al. [37,38] used reinforced ordinary concrete with UHPC and performed mechanical property tests. After roughness treatment, the UHPC-NC specimen had better interface bonding performance and greater interface bonding strength than the specimen without roughness. Zhang et al. [39] carried out an experimental investigation and found that the orientation of the interface had a prominent impact on the bonding strength between existing concrete and self-consolidating concrete. The highest bonding strength was achieved through top bonding. The side and bottom bonding interfaces reduced the bond strength by 6% and 57%, respectively, compared to the top interface.

MARC-ANDR E et al. [40] used UHPC to reinforce R.C. bridge piers with overlapping defects. The results showed that UHPC repair eliminated the splitting of the R.C. bridge pier’s overlapping joints, and the repair effect was excellent. Jiho Moon et al. [41] strengthened an RC plate with UHPC to improve the bending strength and ductility of the plate. After strengthening, the results showed that the ultimate bearing capacity, stiffness, and toughness increased by 70%, 60%, and 128%, respectively.

This study addresses these gaps by preparing recycled brick powder UHPC (RBP-UHPC) through partial cement replacement and assessing its bonding behavior at the interface with frost-damaged concrete. The practical significance of using recycled brick powder in high performance concrete (RBP-UHPC) is its durability and cost effectiveness. The primary purpose is to obtain the best reinforcement method and the change law of bonding performance, which provides a basis for the reinforcement and repair of frost-damaged concrete. Additionally, a multi-factor calculation formula for interface bonding strength was established according to the test data. A micro-mechanism analysis of the RBP-UHPC using scanning electron microscopy (SEM) was carried out, with the aim of gathering data to develop the engineering applications of RBP-UHPC; specifically, there are broad implications in the repair of concrete structures damaged by water freezing, salt freezing, and salt-induced stresses.

## 2. Materials and Methods

### 2.1. Materials

**Figure 1:**
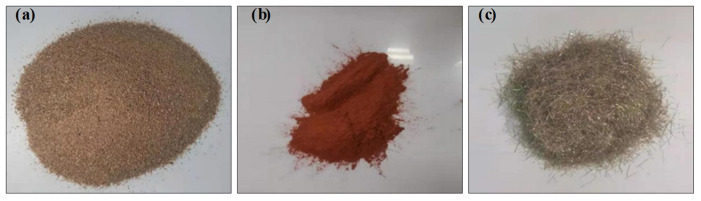
Raw materials used in UHPC: (**a**) river sand; (**b**) recycled brick powder; (**c**) steel fiber.

When producing the RBP-UHPC specimens, ordinary Portland cement (PO_52.5_) produced by Mengdian Cement Industry (Huixian, China) was utilized as the primary binder, and the main components are shown in Table 1. The admixture contained grade I fly ash and Qinghai Yuanheng brand silica ash from Henan Gongyi hannuo filter material Co., Ltd. (Gongyi, China) Fine aggregate was obtained from natural river sand with a particle size ranging from 0.25 mm to 0.5 mm. The main components are shown in Table 2. The coarse aggregate used was a continuous-grade crushed stone obtained from a company in Zhengzhou, with a particle size ranging from 5 mm to 20 mm. The recycled brick powder, a key component, was derived from waste clay bricks collected during demolition and relocation in Zhengzhou City. The compressive strength of the recycled brick powder fell within the range of MU10–MU25, as specified by the “Sintered Ordinary Bricks” (GB/T 5101-2017) standard [42]. To obtain the test recycled brick powder, waste sintered bricks were crushed using a jaw crusher, screened, and ball-milled for 45 min. The main components are presented in Table 3 and shown in Figure 1. Copper-plated steel fibers, 12 mm in length and 0.2 mm in diameter, were supplied by Yutian County Zhitai Steel Fiber Manufacturing Co., Ltd. (Yutian, China). The main components, shown in Table 4, were incorporated. The admixture included a high-efficiency CQJ-JSS polycarboxylic acid water-reducing agent manufactured by Shanghai Chenqi Chemical Technology Co., Ltd. (Shanghai, China), with 26.5% water reduction efficiency. Mixing and curing were conducted using regular tap water sourced from Zhengzhou City. The same cement used in RBP-UHPC, PO_52.5_ OPC, was employed for the interface agent. The swelling agent in the interface agent was the UEA-type expander produced by Wuhan Angsi Manpen Building Materials Co., Ltd., (Wuhan, China). It exhibited a longitudinal limit expansion rate greater than 0.02% after 15 days and a vertical limit drying rate less than 0.02% after 180 days.

### 2.2. Mix Design

**Figure 2:**
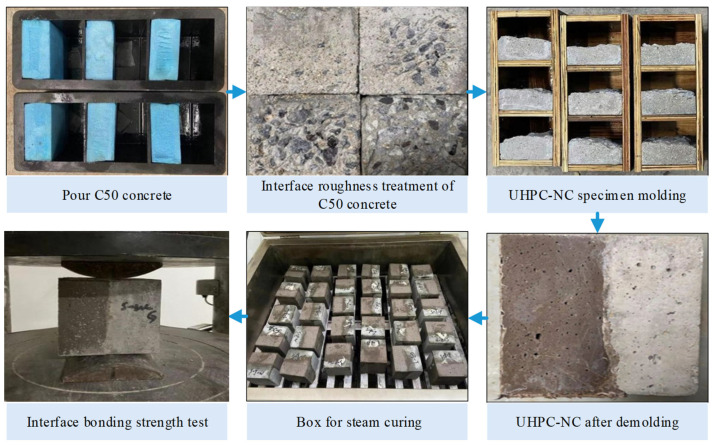
UHPC-NC specimen production and loading process.

By reviewing the literature and adapting the materials, the UHPC mix ratio and 30% RBP-UHPC ratio were obtained; these are presented in Table 5. Figure 2 shows the process of forming the C50 concrete, which was made according to the Design Regulations for Ordinary Concrete Mix Ratio (JGJ 55-2011) standard [43], and the test mix ratio is shown in Table 6. The ratio of the interface agent mix is shown in Table 7.

### 2.3. Specimens Preparing

In the preparation of the UHPC-NC specimens, the methodology drew from the influential work of B.A. Tayeh et al. [44]. The sample preparation process involved the creation of concrete test blocks with specific dimensions, including three sets of C50 concrete test blocks measuring 100 mm × 100 mm × 50 mm; one group of 100 mm × 100 mm × 100 mm × 100 mm blocks were prepared. For the 100 mm × 100 mm × 50 mm blocks, a thorough procedure was followed. Extruded foam panels measuring 100 mm × 100 mm × 50 mm were strategically placed within the mold, and the remaining voids were filled with ordinary C50 concrete. All the above C50 concrete specimens were divided into two parts after 28 days of standard curing, and both parts underwent 100 rapid freeze–thaw cycles in the NaCl solution and water. The data collected showed that the concrete exposed to the NaCl solution experienced the most severe damage at a concentration ranging from 3% to 4%. Therefore, this study used a NaCl solution concentration of 3.5% for the salted tests. After the freeze–thaw cycle, further classification was carried out for the 100 mm × 100 mm × 50 mm blocks. Three distinct groups emerged from this process: Group B1 remained untreated at the interfaces, Group B2 underwent manual gouging, and Group B3 received mechanical gouging. Additionally, a splitting tensile test was conducted after the freeze–thaw cycle for the 100 mm × 100 mm × 100 mm blocks, resulting in cracked interfaces that served as interface roughness treatment (B4).

A measurement method based on the average depth of sand filling was employed to quantify the roughness of the interface surfaces. Four specific ranges were identified: 0.25 mm–0.57 mm, 0.6 mm–1.3 mm, 1.5 mm–2.3 mm, and 3.5 mm–5.3 mm. The treated C50 concrete specimens with varying interface roughness were soaked in water for 12 h, and were then removed and dried. Interface agent treatments were then applied, comprising a no-interface agent (A1), cement net slurry (A2), and cement expansion slurry (A3).

These treatments were performed on the specimens placed in a triple test mold measuring 100 mm × 100 mm × 100 mm, with RBP-UHPC poured sideways. After vibrating and forming for 48 h, specimens were subjected to steam curing in a steam-curing box after removing the molds. After completing these steps, the specimens were transferred to a standard curing room for 28 days. Finally, the UHPC-NC specimens required for the test were obtained, and the YAW-2000B pressure testing machine and prismatic steel block produced in Jinan, China were used for splitting and tensile tests. All the above preparation procedures are detailed in Figure 2.

### 2.4. Experiment Method

The design test procedure for assessing the interface bonding strength of RBP-UHPC-reinforced frozen concrete was carried out in accordance with the specifications “Standard for Test Methods of Long-term Performance and Durability of Ordinary Concrete” (GB/T50082-2009) [45] and “Technical Requirements for Ultra-high Performance Concrete” (T/CECS 10107-2020) [46]: the final size of the cubes was 100 mm × 100 mm × 100 mm, and the working conditions were water freezing and salt freezing. When the interface agent was applied, it was divided into three methods: no interface agent, cement paste, and cement paste slurry. The three interfaces were labeled A1, A2, and A3, respectively. The interface roughness treatment was divided into four methods: no treatment, artificial chiseling method, mechanical chiseling method, and splitting method. These treatments have four interface types, identified as B1, B2, B3, and B4, respectively. In the experimental design, the specimens were subjected to varying numbers of freeze–thaw cycles: 0, 50, 100, and 150 freeze–thaw cycles. The primary criterion for evaluating the performance of these specimens was the interface bonding strength.

**Figure 3:**
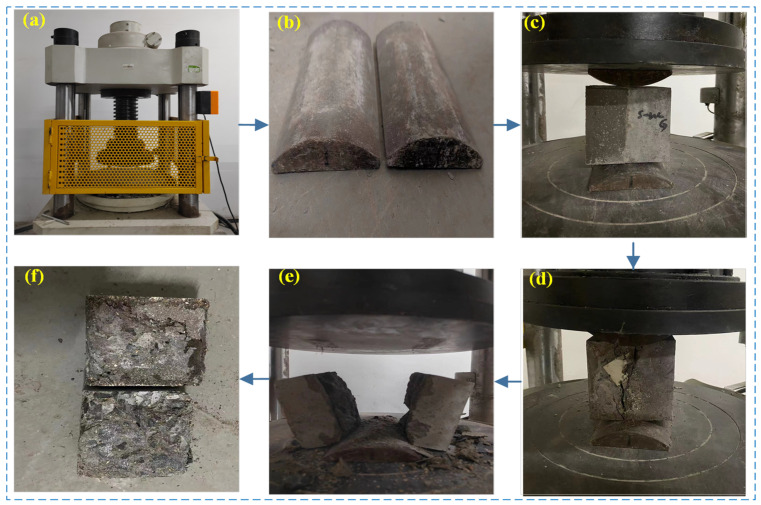
Experimental setup: (**a**) YAW-2000B/300C testing machine; (**b**) prismatic steel block; (**c**) specimen before load application; (**d**) specimen initial cracking; (**e**) specimen splitting failure; (**f**) specimen after testing.

According to the “Technical Requirements for Ultra-high Performance Concrete” (T/CECS 10107-2020) [46] and the Standard for Test Methods for Mechanical and Physical Properties of Concrete (GB/T 50081-2019) [47], the interface bonding strength test, specifically the splitting tensile strength test, was carried out on the UHPC-NC specimens. The testing equipment comprised a YAW-2000B pressure testing machine, a prismatic steel block, and an experimental setup designed to ensure precise measurements and reliable results, as illustrated in Figure 3.

The interface bonding strength $f_{t}$ (MPa) of the UHPC-NC specimens was determined through testing. The relative index *β*_n_ for splitting tensile strength and the index *γ*_n_ for the rate of decrease in splitting tensile strength were introduced. *β*_n_ represents the ratio of splitting tensile strength in the UHPC-NC specimens to that in ordinary C50 concrete after *n* freeze–thaw cycles.

*γ*_n_ represents the ratio of splitting tensile strength in the UHPC-NC specimens after *n* freeze–thaw cycles to the splitting tensile strength in the UHPC-NC specimens without any freeze–thaw cycles.

(1) $$\beta_{n}=\frac{f_{tsn}^{UN}}{f_{ts}^{N}}\times 100\%$$

(2) $$\gamma_{n}=\frac{f_{tsn}^{UN}}{f_{ts0}^{UN}}\times 100\%$$

where $f_{tsn}^{UN}$ represents the splitting tensile strength (MPa) of the UHPC-NC bonding specimen after *n* freeze–thaw cycles; $f_{ts}^{N}$ represents the splitting tensile strength of ordinary concrete without freeze–thaw cycles; $f_{ts0}^{UN}$ represents the UHPC-NC splitting tensile strength of bonding specimens without freeze–thaw cycles.

### 2.5. Test Phenomena

**Figure 4:**
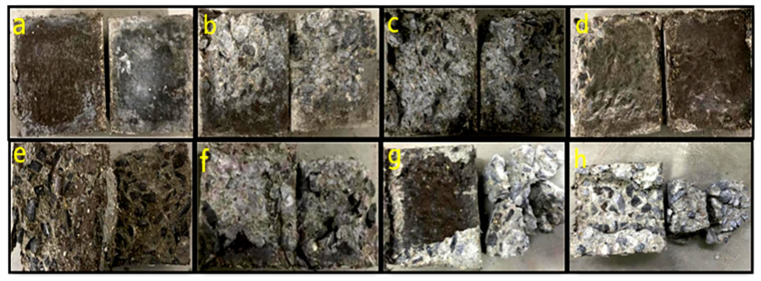
UHPC-NC specimens interface destruction types: (**a**) failure with minimal adhesion and smooth surface; (**b**) failure with limited steel fibers and fragment attachment; (**c**) steel fibers and frost-damaged concrete; (**d**) interface agent destruction; (**e**) interface agent and concrete damage; (**f**) concrete failure and agents; (**g**) failure with crushed concrete, gravel, and fibers; (**h**) failure with crushed concrete with agents.

The interface bonding of the UHPC-NC specimens is the weakest part. The splitting tensile test specimen failure is usually near this position. Its failure location and failure form are related to many factors. By observing the interface bonding failure state, UHPC-NC specimen interface failure can be divided into the following types according to freeze–thaw cycle numbers [48], as illustrated in Figure 4. Class A failure is characterized by a bonding surface failure involving frost-damaged concrete and UHPC, resulting in minimal material adhesion and a smooth surface. Class B represents bonding surface failure between UHPC and ordinary concrete, with a limited number of steel fibers protruding from the UHPC bonding surface, with some frost-damaged concrete fragments attached to it, and some aggregates peeled off at the frost-damaged concrete bonding surface.

Class C failure affects ordinary concrete failure without an interface agent. It is characterized by steel fibers protruding from the UHPC bonding surface, substantial attachment of frost-damaged concrete, significant damage to the frost-damaged concrete bonding surface, and some fibers adhering to it. Class D failure indicates the destruction of the interface agents, as evidenced by the presence of interface agents attached to the frost-damaged concrete and UHPC failure surfaces, along with a limited amount of steel fibers protruding at the UHPC interface bond. Class E failure is the destruction of interface agents and ordinary concrete, which is manifested by the adhesion of interface agents on the surface of frost-damaged concrete and UHPC, and the steel fiber protruding and partial frost-damaged concrete aggregate attached to the surface of UHPC. Class F failure is the failure of ordinary concrete when there is an interface agents, which is manifested as the fracture position of the specimen is on the side of ordinary concrete; here, there is an interface agent and a large amount of concrete crushed stone adhered to the surface of UHPC, the ordinary concrete bonding surface is seriously damaged, and a large amount of aggregate is exposed. Class G failure is failure without the interface agents; the concrete is crushed, which is manifested as concrete gravel and steel fibers adhering to the UHPC side, and the ordinary concrete damage has been crushed if it is serious. Class H failure is failure when there is an interface agent; the concrete is crushed, which manifests as an interface agent on the UHPC side, the concrete gravel is attached to it, and the ordinary concrete side has been crushed.

Generally, when the failure location is nearer to the regular concrete side, the bonding effect tends to be better due to higher interface bonding strength. Conversely, when failure occurs directly at the bonding surface, the bonding effect is weaker, resulting in the lowest splitting strength for the reinforced specimen.

## 3. Test Results and Analysis

### 3.1. Interface Bonding Properties of RBP-UHPC-Reinforced Frost-Damaged Concrete under Water Freezing Conditions

**Figure 5:**
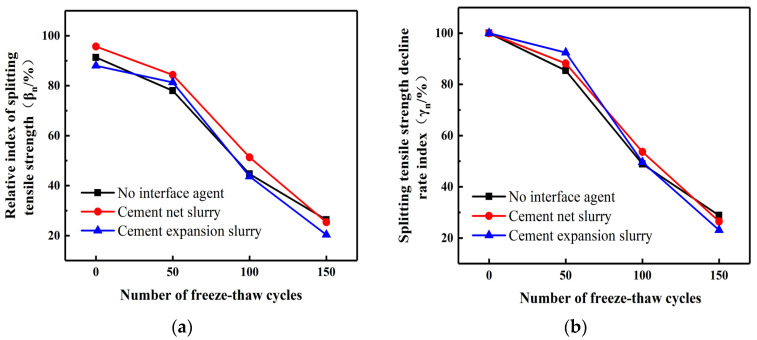
Effects of interface agents on different indices of splitting tensile strength of water frozen specimens. (**a**) Relative index of splitting tensile strength. (**b**) Splitting tensile strength decline rate index.

This study conducted splitting tensile strength tests on UHPC-NC test blocks after water freezing and calculated the corresponding *β*_n_ and *γ*_n_. Analyzed the effects of interface agents and roughness on the splitting tensile strength of specimens under water freezing cycles. Figure 5. provides insight into the early stages of water freezing cycles, showing the splitting strength of UHPC-NC specimens coated with cement net slurry, which exhibited the most significant relative index increase. Conversely, UHPC-NC specimens with cement expansion slurry displayed the highest splitting tensile strength index.

The use of interface agents was observed to enhance the interface bonding strength of the reinforced specimens. This enhancement was primarily attributed to numerous pores and microcracks within the bonding interface, resulting from frost damage to the ordinary concrete. Without interface agents, the cement slurry in UHPC could only partially fill the voids within the ordinary concrete. However, after applying the interface agents, the interface agents can make the hydration reaction of cement more complete. The hydration reaction products will grow into these holes and microcracks, filling most of the holes in ordinary concrete and increasing the mechanical bite force between the interfaces. The influence of the interface agents on the specimen decreases with an increase in the freezing cycles. By the end of the hydro-freezing cycle, the splitting tensile strength decreases at a rate that is the same or even weaker than that of the specimen without the interface agents. This is because other materials, such as steel fibers in UHPC, will reduce their shrinkage so that there is a shrinkage difference between UHPC and NC, resulting in tensile and compressive stress. This is superimposed with the tensile compressive stress caused by temperature deformation during the hydro-freezing cycle, resulting in a decrease in bonding strength and the inability of the interface agents to play an important role [49]. Therefore, cement expansion as an interface agent should be minimized in harsh environments such as low-temperature environments.

**Figure 6:**
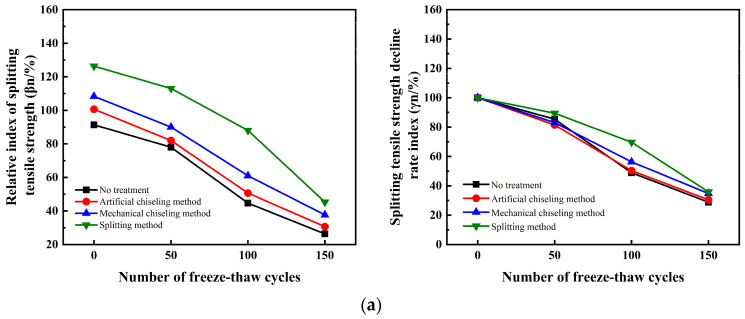

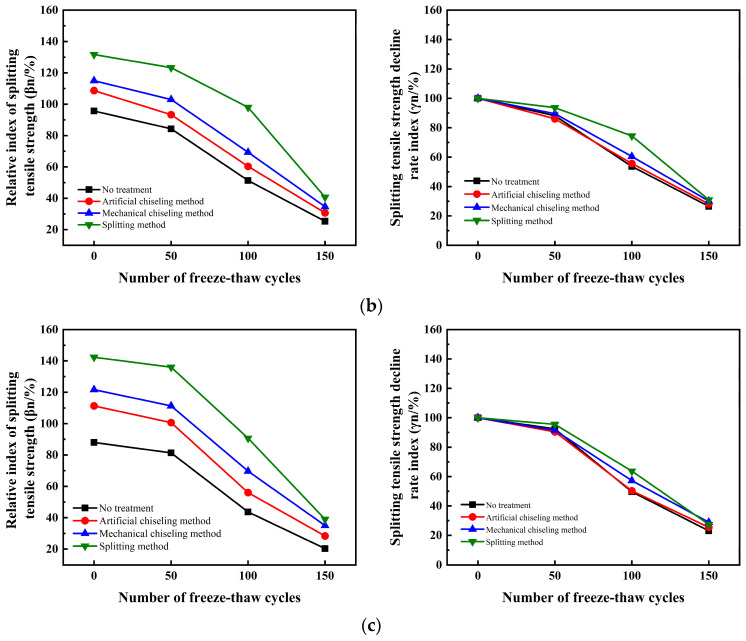
Effect of interface roughness on the relative index of splitting tensile strength and strength decline rate index of hydro-frozen specimen. (**a**) No interface agent. (**b**) Cement net slurry. (**c**) Cement expansion slurry.

Figure 6 illustrates the impact of various interface roughness treatment methods on the decrease rate of split tensile strength index *γ*_n_ of UHPC-NC specimens; this impact is less than the effect on the relative index *β*_n_ of split tensile strength. Initially, the hydro-freezing cycle of the splitting strength of the specimen under the four interface roughness treatments aligns with the *β*_n_ curve. However, during the later stage of the hydro-freezing cycle, the splitting strength of UHPC-NC specimens treated by the splitting method at the interface decreased the fastest relative index *β*_n_ because the NC in the specimen was frost-damaged concrete. When the hydro-freezing cycle test was repeated, the performance worsened, resulting in its strength declining faster and faster. In the initial stage of the hydro-freezing cycle, the specimen subjected to the roughness method showed the highest splitting tensile strength with the smallest reduction rate (*γ*_n_), and its slope was the smallest. As the number of freezing cycles increased, the reinforced specimens treated with the four roughness methods displayed a pattern of initially rising and subsequently declining in the rate of reduction for their splitting tensile strength. After 150 hydro-freezing cycles, splitting- and pulling-method-treated specimens decrease at splitting tensile strength *γ*_n_ rate, while the other three roughness treatments are closer and closer, and its slope becomes the largest. Increasing freezing cycles degrade the C50 concrete state, decreasing the splitting strength of the UHPC-NC specimens. The splitting strength of the reinforced specimen becomes smaller and smaller; the impact of the interface roughness treatment method on the splitting strength becomes smaller and smaller; the specimen treated with the splitting method gradually loses its advantage; the splitting tensile strength decreases faster and faster. This leads to an acceleration in the splitting strength reduction rate index; the reinforcement specimens under the other three kinds of interface roughness treatment become increasingly similar.

### 3.2. Interface Bonding Properties of RBP-UHPC-Reinforced Frost-Damaged Concrete under Salt Freezing Conditions

**Figure 7:**
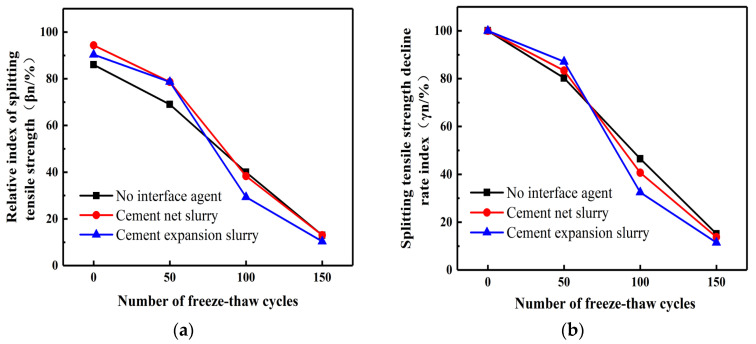
Effect of interface agents on different indices of splitting strength of salt frozen specimens. (**a**) Relative index of splitting tensile strength. (**b**) Splitting tensile strength decline rate index.

The splitting tensile strength test was carried out after exposing the UHPC-NC test block to salt freezing; during testing, the corresponding *β*_n_ and *γ*_n_ values were measured. Salt freezing cycles ranged from 0 to 50; the relative index of the splitting tensile strength in the cement paste specimens was higher than that of specimens without an interface agent. These showed 14.01% and 14.97% higher values, respectively, as shown in Figure 7. The relative index of the splitting tensile strength for cement paste specimens was 14.01% for those with an interface agent, while specimens without an interface agent displayed a relative index of 14.97%. This finding highlights the beneficial role of interface agents in improving tensile strength after salt freezing. However, as salt freezing cycles increased, the positive impact of the interface agent diminished, and the tensile strength of the specimens was increasingly affected. This indicates that prolonged exposure to salt freezing conditions reduces the effectiveness of the interface agent. Remarkably, at 100 cycles of salt freezing, the cement expansion slurry interface agent demonstrated its maximum effectiveness, as it resulted in the smallest relative index of splitting tensile strength and the least reduction in tensile strength in comparison with the other specimens.

In contrast, specimens without an interface agent exhibited the highest reduction in tensile strength at this stage. This result highlights the pivotal role of the cement expansion slurry and interface agent in maintaining tensile strength at this specific salt freezing cycle count. At 150 cycles of salt freezing, the two indices under three different interface agents are almost the same. Because the interface agent is greatly affected by salt freezing, the cement slurry interface agent is more affected by salt freezing, and the salt freezing cycle accelerates the failure rate of the reinforced specimen.

**Figure 8:**
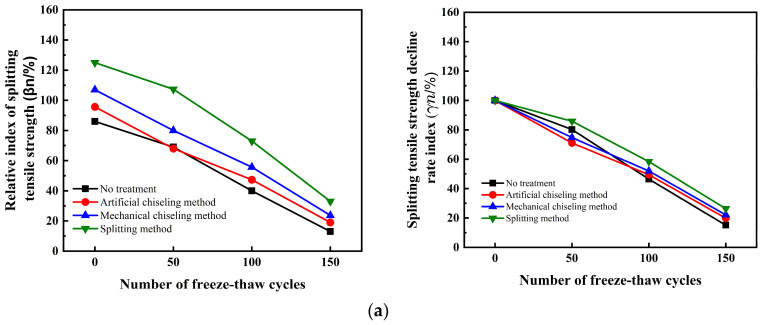

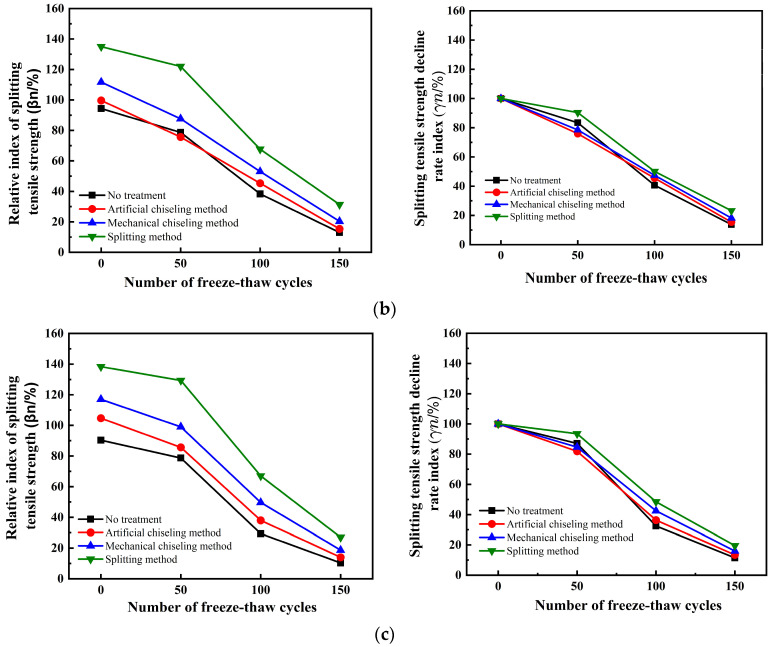
Effect of interface roughness on the relative index of splitting tensile strength and strength decline rate index of hydro-frozen specimens. (**a**) No interface agent. (**b**) Cement net slurry. (**c**) Cement expansion slurry.

According to the findings presented in Figure 8, the influence of interface roughness on the splitting tensile strength of UHPC-NC specimens subjected to salt freezing cycles is noteworthy. It is evident that interface roughness substantially impacts the relative *β*_n_ index of the interface bond splitting tensile strength, especially in the early stages of the salt freezing cycle. However, the influence of interface roughness on the *γ*_n_ index of splitting tensile strength is limited, and this effect becomes even more negligible as the salt freezing cycles progress. The decline rate indices of the splitting tensile strength of the reinforced specimens under four kinds of roughness were found to be very close. In the early stages of the salt freezing cycle, the two indices of the specimen obtained by the splitting method were found to be significantly better than those of the other three methods. After 150 salt freezing cycles, the two indices of the four interface roughness treatment methods become smaller and closer, within 10%~30%; this is less than the corresponding index of the UHPC-NC specimen underwater freezing cycle. This phenomenon can be attributed to the fact that, as the count of freeze–thaw cycles escalates, the salt solution’s water retention also escalates, resulting in a heightened water saturation within the concrete. This elevated saturation intensifies the damage caused by salt freezing in the concrete. The freezing of the solution within the pores generates concealed stresses inside the concrete, which eventually lead to substantial internal damage; this observation corroborates previous experimental research carried out by Tahmureszadeh, K. et al. [50].

In terms of the overall freeze–thaw cycle process, both indices of UHPC-NC specimens treated by splitting method are far higher than those of ordinary C50 concrete. The splitting tensile strength is also far higher than the splitting tensile strength of frozen C50 concrete specimens, and the reinforcement effect is good.

## 4. UHPC-NC Interface Bonding Strength Calculation Method

According to the previous test data analysis, a method for evaluating the interface bonding strength between RBP-UHPC and frozen concrete is proposed, which provides theoretical guidance for practical engineering applications.

Researchers have introduced approaches to determine bonding strengths at the interfaces between newly placed and preexisting concrete. Wang Mengwei [51] established coefficients for various influencing variables and formulated an equation to compute the bonding strength at the interface of ECC and preexisting concrete. Gao Zichen [52] introduced a method for evaluating the interface bonding strength between lightweight aggregate concrete and regular concrete.

### 4.1. Calculation Formula of UHPC-NC Interface Bonding Splitting Strength at Room Temperature

In this paper, through studying the influencing factors such as roughness, interface agent, and freeze–thaw conditions, we concluded that the interface bond strength of UHPC-NC is affected equally by the roughness, the interface agent, and the freeze–thaw conditions. According to the content of previous research, the multi-factor calculation formula of interface bonding strength at room temperature was established:

(3) $$\tau (f_{t})=\alpha_{1}\phi_{1}\phi_{2}\phi_{3}$$

In the formula, *τ* represents the interface bond strength; $\alpha_{1}$ is the fitting coefficient (MPa); $\phi_{1}$ is the impact coefficient of the interface agent (dimensionless); $\phi_{2}$ is the roughness influence coefficient (dimensionless); $\phi_{3}$ is the coefficient of influence of freeze–thaw conditions (dimensionless).

**Figure 9:**
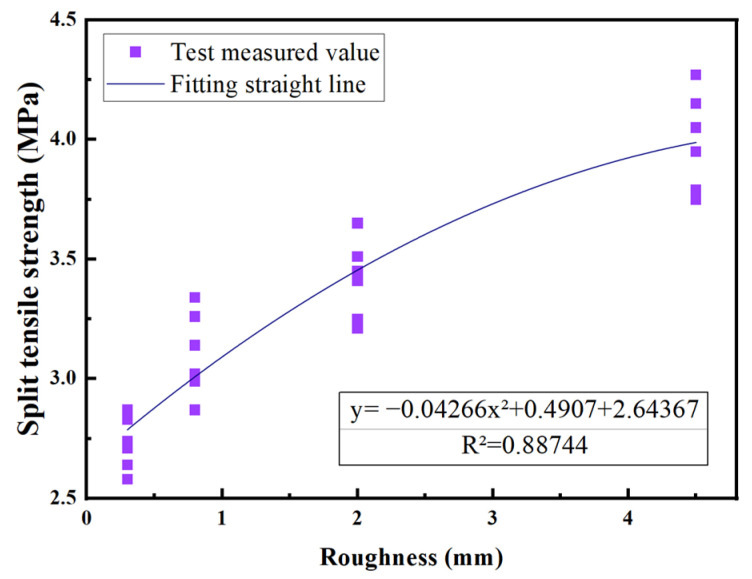
Fitting of each data point under different roughness indices.

Based on the ratio of splitting tensile strength observed in specimens using different interface agents, the ratios are as follows: when no interface agent is present, the ratio $\phi_{1}$ = 1; when the interface agent is cement paste, $\phi_{1}$ = 1.079; and when the interface agent is cement slurry, $\phi_{1}$ = 1.106. Using the collected test data, a linear regression equation was employed to assess the impact of interface roughness on the interface bonding strength of the UHPC-NC specimens. The analysis curve is shown in Figure 9.

The test data were analyzed through a regression experiment, and the relationship between splitting tensile strength and roughness was obtained as follows:

(4) $$y=-0.04266x^{2}+0.04907x+2.643$$

The determination coefficient R^2^ was found to be 0.85846, and the fitting was found to be good. Therefore,

(5) $$\phi_{2}=-0.04266h^{2}+0.04907h+2.643$$

According to the ratio of splitting tensile strength of specimens under water freezing and salt freezing environments, the following were found to be true: when the working condition is water freezing, $\phi_{3}$ = 1; when the working condition is salt freezing, $\phi_{3}$ = 1.062.

**Figure 10:**
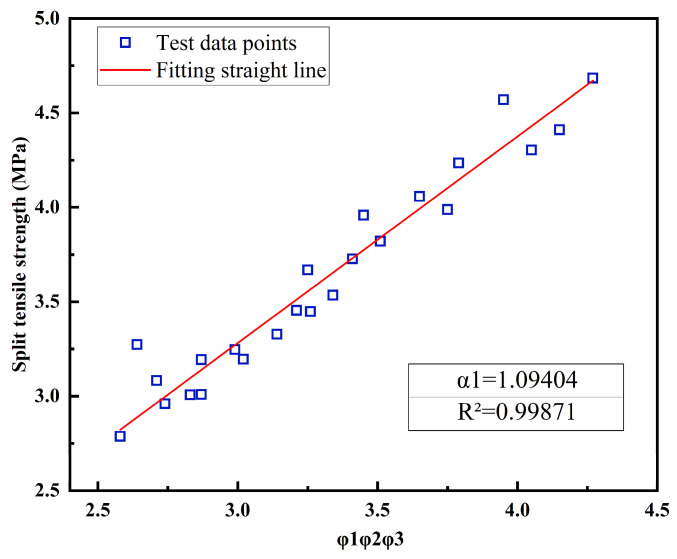
The combined effects of “*φ*_1_*φ*_2_*φ*_3_” on the interface bonding strength.

Through an examination of various factors affecting the interface bond strength of UHPC-NC specimens, we determined the combined effect of the interface agent, the roughness, and the freeze–thaw conditions on the interface bond splitting tensile strength; the results are depicted in Figure 10. It can be seen from Figure 10 that the fitting coefficient is 1.094, R^2^ is 0.998971, and the fitting line is very close to the experimental value. 

Finally, combined with all the data, the calculation formula of bond splitting tensile strength of the UHPC-NC interface was performed, as follows:

(6) $$f_{t}=1.162\phi_{1}\phi_{3}(-0.04266h^{2}+0.04907h+2.643)$$

### 4.2. Formula for Evaluating Interface Bond Strength of UHPC-NC during Freeze–Thaw Cycling

**Figure 11:**
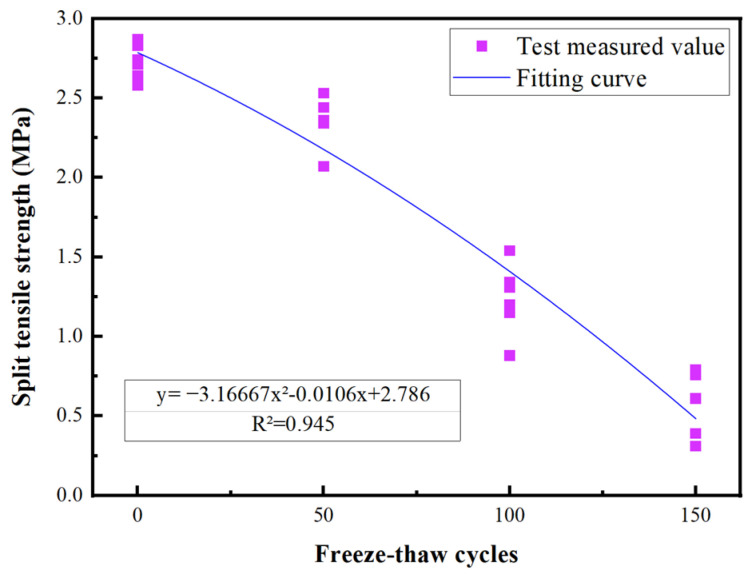
Fitting of each data point under different roughness.

According to the strength calculation formula under the freeze–thaw cycle state, the multi-factor calculation formula of interface bonding strength in the freeze–thaw cycle state was introduced. In this formula, $\omega_{1},\omega_{2},\omega_{3}$ are the same as $\phi_{1},\phi_{2},\phi_{3}$, respectively; $\omega_{4}$ represents the coefficient of influence of the number of freeze–thaw cycles (dimensionless). According to the test data obtained above, the linear regression equation was used to analyze the influence of the number of freeze–thaw cycles on the interface bonding strength of UHPC-NC specimens, and the analysis curve is shown in Figure 11.

(7) $$\tau (f_{t})=\alpha_{2}\omega_{1}\omega_{2}\omega_{3}\omega_{4}$$

Regression analysis used to validate experimental data, it can be concluded that the relationship between the tensile strength of splitting and the number of freeze–thaw cycles is:

(8) $$y=-3.1667x^{2}-0.0106x+2.786$$

The determination coefficient R^2^ was found to be 0.945, and the fitting was found to be good. Therefore,

(9) $$\omega_{4}=-3.1667\times 10^{-5}n^{2}-0.0106n+2.786$$

**Figure 12:**
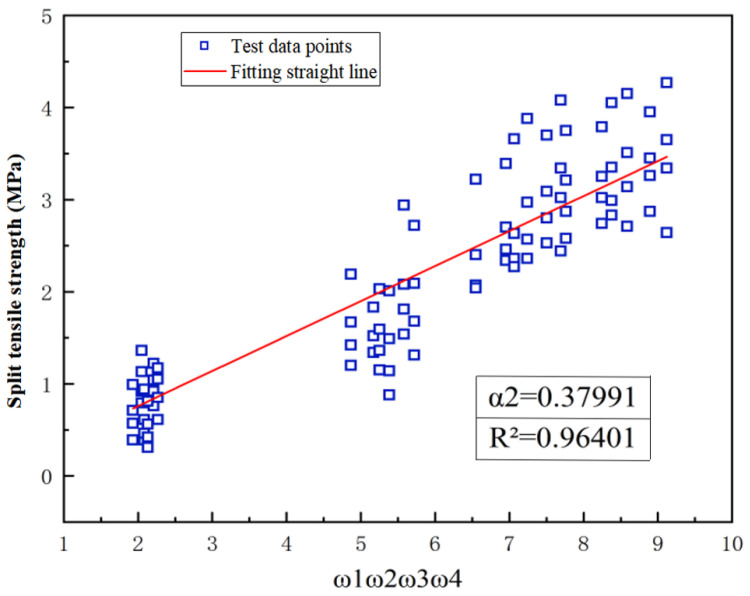
The combined effects of “ω_1_ω_2_ω_3_ω_4_” on the interface bonding strength.

Through assessing the influence of different factors on the interface bonding strength of the UHPC-NC specimens, the comprehensive impact of the interface agent, the roughness, and the freeze–thaw conditions on the interface bonding splitting tensile strength is depicted in Figure 12. It can be seen from Figure 12 that the fitting coefficients are 0.37991 and 0.96401, and the fitting line is very close to the experimental value. Finally, combining all the data, the formula for calculating the bonding and splitting strength of the UHPC-NC interface under the final freeze–thaw cycle is as follows:

(10) $$\begin{array}{c} f_{t}=0.37991\omega_{1}\omega_{3}\times (0.04266h^{2}-0.04907h-2.643)\\ \times (3.1667\times 10^{-5}n^{2}+0.0106n-2.786) \end{array}$$

## 5. Bonding Mechanism and Model Analysis

The interface bonding mechanism between RBP-UHPC and frost-damaged C50 concrete was studied by an SEM microscopic test to analyze its microscopic characteristics. The UHPC-NC bonding interface was scanned by high-magnification light microscopy, the state of the two materials at the bonding interface was analyzed, and the UHPC-NC bonding model was established.

### 5.1. SEM Test

A cube test block with a length of 10 mm was taken on the bonding interface of UHPC-NC and observed using scanning electron microscopy (SEM) and the micromorphology of the UHPC-NC specimen interface under the three different interface agent treatments. The goal was to gain insights into the arrangements and distributions of various components, including the recycled brick powder, the cementitious matrix, and any other relevant constituents.

**Figure 13:**
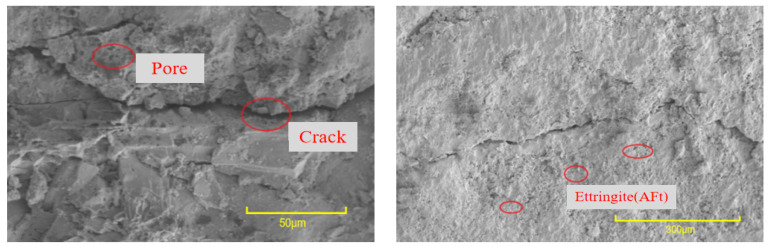
Micromorphology of interfaces bonded without interface agent.

**Figure 14:**
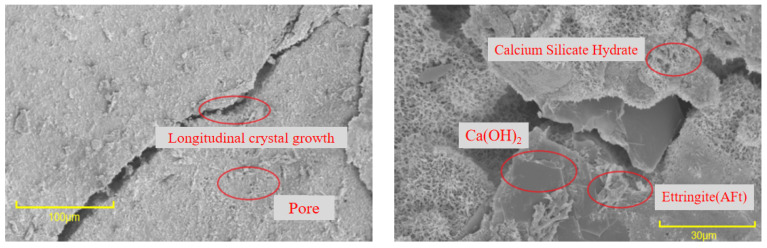
Micromorphology of the adhesive interface of applying cement net slurry.

**Figure 15:**
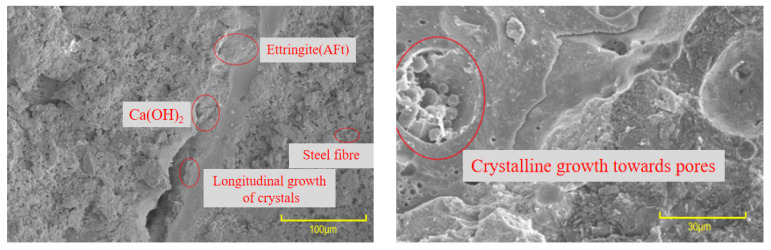
Micromorphology of the adhesive interface of applying cement expansion slurry.

It can be seen from Figure 13 that, where no interface agent was used, significant cracks were evident at the bonding interface. These cracks were primarily attributed to inadequate cement hydration, resulting in the formation of only a limited number of AFt crystals and an overall poor compactness of the microstructure. In contrast, Figure 14 and Figure 15 illustrate a remarkable improvement after applying the interface agent. The cracks at the bonding interface are significantly smaller. The wetting degree of the old concrete surface increased, and the water–cement ratio increased, so the cement hydration degree was relatively sufficient. At this time, a large number of crystals formed at the bonding interface, such as hydrated calcium silicate, calcium hydroxide, and ettringite (AFt). These crystals exhibited intricate interwoven patterns and longitudinal growth along the interface, with some even penetrating the concrete voids, further enhancing the microstructure’s compactness. The enhanced compactness of the microstructure correlated with an improvement in the interfacial bonding strength of the UHPC-NC specimens. In summary, this SEM analysis highlights the significance of interface agent treatments in the improving microstructural integrity and the interfacial bonding strength of the specimens, holding promising potential for the repair and reinforcement of concrete structures subjected to freezing- and salt-induced stresses; these findings are similar to those presented in the conclusions of previous research by Zhang Y. et al. [53].

### 5.2. Bonding Model Analysis

Regarding the model of the interface bonding between preexisting and newly placed concrete, the widely acknowledged framework is the three-zone bonding model, which was introduced by Emmnos et al. [54]. The model incorporates three separate zones: the upper old concrete zone, the intermediate interface zone, and the lower new concrete zone.

Lyubmiov [55] introduced the idea of a transition region, which partitioned the boundary between cement stone and aggregate into three sections: a diffusion layer, a strong effect layer, and a weak effect layer. Among them, the diffusion layer is close to the aggregate side, which has a weak effect on the performance of the interface. The strong effect layer comprises cement hydration products such as alumina, calcium hydroxide crystals, and C-S-H gels. There are fewer hydration products in the weak effect layer, and the impact on the interface properties is small.

This research shows that the mechanical performance of the transition layer is poor, and the damage is severe. The thickness is generally 10–50 μm. The microscopic morphologies and mechanical properties of the transition layer determine the success of the reinforced specimen. The transition layer is the weak layer of the whole bonding area, and stress damage usually occurs in the transition layer. When we study the interface failure characteristics of the reinforced specimen, it can be attributed to the study of the failure performance of the transition layer concrete.

**Figure 16:**
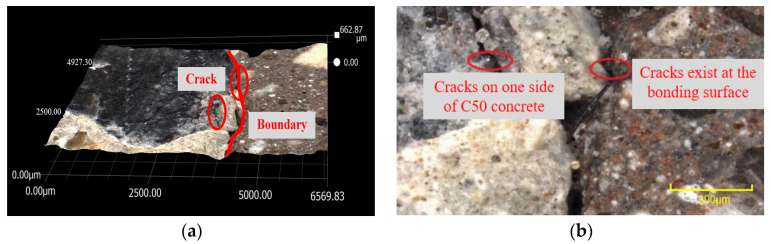
Magnified optical microscopy images of UHPC-NC bonding interface without interface agent. (**a**) Magnified 50 times. (**b**) Magnified 200 times.

The bonding surface of the specimen is scanned by a high-magnification optical microscope, as shown in Figure 16. When the bonding interface is magnified 50 times and 200 times, it can be seen that there are microcracks on the ordinary concrete side near the bonding interface. Cracks are visible at the bonding interface. The mechanical properties of concrete in the presence of cracks are poor, indicating that the transition layer near the concrete side is the weak layer of the UHPC-NC specimens. The interface bonding model of the UHPC-NC specimens without an interface agent is a three-zone model, and the interface-agent-affected zone consists of three parts: the old concrete–interface agent transition layer (L1), the interface agent layer (L2) and the interface agent–UHPC transition layer (L3). The formation mechanism of the old concrete–interface agent transition layer L1 is as follows: When the interface agent is applied to the surface of the old concrete, the water–cement ratio in the transition layer becomes higher than that in the interface agent and UHPC. This high water–cement ratio increases the ettringite and calcium hydroxide crystals at the interface, which is the main component of the transition layer. In addition, the interface agent and some active sodium and calcium ions in the UHPC also penetrate the old concrete. Some active ions will react with the cement stone in the old concrete, which is also part of forming the L1 transition layer. With the same type and thickness of interfacial agent, as concrete ages, its porosity increases, resulting in a higher water–cement ratio in ultra-high-performance concrete (UHPC) and a thicker transition layer.

**Figure 17:**
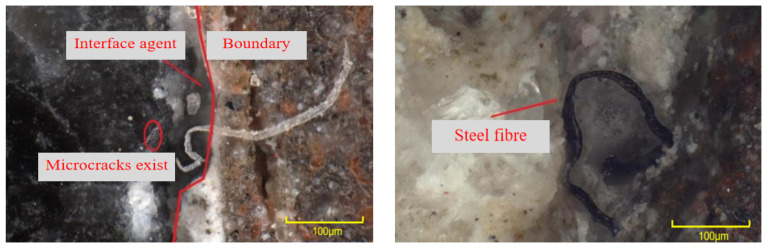
The UHPC-NC bonding interface was magnified 500 times after applying the interface agent.

The interface agent layer L2 is composed of the interface agent itself, and its performance is also closely related to the UHPC and ordinary concrete present. The interface agent is generally only about 3 mm deep. During the pouring and hydration process, some sodium ions and calcium ions in the UHPC will enter the interface agent layer, and the steel fiber in the UHPC will also enter the transition layer. The UHPC-NC interface agent layer can be observed through an optical microscope. The presence of fibers in the interface agent can be seen, as shown in Figure 17. The above shows that the interface agent has become a transition layer in the process of vibration hydration.

The interface agent–UHPC bonding transition layer L3 is a gradual transition layer from the newly poured UHPC to the interface agent. The number and shape of crystals in this area are similar to those of the UHPC. However, due to the simultaneous vibration and hydration with the interface agent and the presence of fibers in the UHPC, the layer is closely bonded to the interface agent, which has little effect on the performance of the interface. It can be predicted that a three-zone–three-layer bonding model can characterize the bonding characteristics of the UHPC-NC specimens coated with interface agents.

## 6. Conclusions

(1) Based on the findings of this study, the 30% recycled brick powder replacement rate into UHPC shows significant improvement in durability and frost resistance. The strength and relative dynamic elasticity modulus remained unchanged even after 150 freeze–thaw cycles. Therefore, the recycled brick powder UHPC can form a good adhesion with ordinary concrete, making it a valuable option for reinforcing existing frost-damaged concrete in cold areas.
(2) During the initial phase of the freeze–thaw cycle, the specimens treated with the interface agent show stronger bonding at the interface as compared to specimens without the agent. However, as the number of freeze–thaw cycles increased, the beneficial impact of the interface agent on the specimens diminished gradually.
(3) The bonding strength at the interface of the UHPC-NC specimens is weaker during salt freezing cycles compared to water freezing cycles. Additionally, the rate of decline in bonding strength is faster in the salt freezing environment, indicating more severe damage to the UHPC-NC specimens.
(4) This study used a multi-factor formula and linear regression equation to accurately predict interface bonding strength in the UHPC-NC specimens based on factors like the interface agents used, the roughness, and the freeze–thaw conditions, aligning well with experimental results showing its accuracy.
(5) Microscopic analysis of the bonding interface revealed that the bonding interface was not coated with the interface agent and showed poor bonding, due to the absence of the interface agent, large cracks, and insufficient cement hydration in the UHPC-NC. A three-zone–three-layer bonding model provides valuable insights into the intricate bonding mechanism between UHPC and NC.

## Figures and Tables

**Table 1:** Main technical indices of cement.

Specific Surface Area (m^2^·kg^−1^ )	Stability	SO_3_ (%)	Cl^−^ (%)	Admixture (%)	Ignition Loss (%)	Setting Time (min)		28-Day Strength /MPa	
						Initial Set	Final Set	Flexural Strength	Compressive Strength
386	Qualification	2.33	0.036	6.0	2.30	170	222	7.5	55.6

**Table 2:** Particle size distribution of river sand.

Grain Size (mm)	<0.075	0.075–0.17	0.17–0.315	0.315–0.63	0.63–1.25	>1.25
Content/%	0.67	3.37	10.00	36.12	45.35	4.49

**Table 3:** Main indices and components of RBP.

Specific Surface Area (m^2^·kg^−1^)	Moisture Content (%)	Major Contents of Components (%)				
		SiO_2_	Al_2_O_3_	Fe_2_O_3_	CaO	MgO
600	≤0.50	67.83	16.20	7.55	1.67	0.94

**Table 4:** Main technical indices of steel fiber [25].

Length/mm	Diameter/mm	Density/g·cm^−3^	Form	Tensile Strength/MPa
12.0	0.2	7.8	Formed straight and smooth	>2000.0

**Table 5:** RBP-UHPC mix ratio (kg/m^3^).

Constituencies	Substitution Rate/%	Recycled Brick Powder	Water	Fly Ash	Steel Fiber	Cement	Silica Ash	River Sand	Water Reducer
R0	0	0	160	100	156	700	200	1000	30
R1	30	210	160	100	156	490	200	1000	30

**Table 6:** C50 concrete mix ratio (kg/m^3^).

Constituencies	Sand	Cement	Macadam	Water
NC	556	476	1183	195

**Table 7:** Interface agents mix ratio (kg/m^3^).

Constituencies	Interface Agent Name	Material Ratio	Water-to-Adhesive Ratio
A1	No interface agent	-	-
A2	Cement net slurry	Cement: water = 1:0.4	0.04
A3	Cement expansion slurry	Cement: Water: Bulking Agent = 1:0.29:0.11	0.04

## Data Availability

We would like to declare that the data employed and analyzed during the current study will be available from the corresponding author upon request, and we are committed to providing access to our data to ensure the transparency and reproducibility of our findings.
